# Constructing a Concentric GO Network via Rotational Extrusion for Synergistic Axial–Hoop Mechanics in Polymer Microtubes

**DOI:** 10.3390/polym18020273

**Published:** 2026-01-20

**Authors:** Wenyan Wang, Wen Liang, Guanxi Zhao, Rui Han, Min Nie

**Affiliations:** 1 State Key Laboratory of Advanced Polymer Materials, Polymer Research Institute of Sichuan University, Chengdu 610065, China; wwyandmmy@163.com (W.W.);; 2 School of Materials Science and Engineering, Key Laboratory of Materials and Surface Technology (Ministry of Education), Engineering Research Center of Intelligent Air-Ground Integration Vehicle and Control, Xihua University, Chengdu 610039, China; 3 Sichuan Special Equipment Inspection Institute, Technology Innovation Center of Hydrogen Storage-Transportation and Fueling Equipments, State Administration for Market Regulation, Chengdu 610000, China

**Keywords:** PLA microtubes, rotational extrusion, mechanical properties

## Abstract

Driven by societal and technological progress, the polymer tubing industry is increasingly focused on sustainable and biodegradable products, with polylactic acid (PLA)-based microtubes gaining attention for applications such as medical stents and disposable straws. However, their inherent mechanical limitations, especially under hoop loading and the brittleness of PLA, restrict broader use. Although two-dimensional nanofillers can enhance polymer properties, conventional extrusion only creates uniaxial alignment, leaving fillers randomly oriented in the radial plane and failing to improve hoop performance. To address this, we developed a rotational extrusion strategy that superimposes a rotational force onto the conventional axial flow, generating a biaxial stress field. By adjusting rotational speed to regulate hoop stress, a concentric, interlocked graphene oxide network in a PLA/polybutylene adipate terephthalate microtube is induced along the circumferential direction without disturbing its axial alignment. This architecturally tailored structure significantly enhances hoop mechanical properties, including high compressive strength of 0.54 MPa, excellent low-temperature impact toughness of 0.33 J, and improved bending resistance of 30 N, while maintaining axial mechanical strength exceeding 50 MPa. This work demonstrates a scalable and efficient processing route to fabricate high-performance composite microtubes with tunable and balanced directional properties, offering a viable strategy for industrial applications in medical, packaging, and structural fields.

## 1. Introduction

Driven by societal and technological progress, the polymer tubing industry is transforming, with a growing emphasis on sustainable and biodegradable microtube products [1,2]. Consequently, biodegradable microtubes—especially those based on polylactic acid (PLA)—have attracted considerable attention [3,4]. PLA-based versions are already commercially available in products such as medical interventional stents, functional catheters, and disposable food-grade straws [5,6]. However, polymer microtubes inherently suffer from insufficient mechanical performance due to their small-sized hollow structure, particularly prone to deformation, collapse, or even rupture under hoop loading [7,8,9]. Furthermore, the intrinsic brittleness of PLA often results in inferior mechanical strength compared to conventional polymer microtubes [10]. Nanofiller reinforcement is an effective approach to improving the mechanical properties of polymers [11,12]. Compared with one-dimensional fibrous fillers, two-dimensional sheet-like fillers possess a high aspect ratio, which not only enables bidirectional reinforcement of the polymer, but also significantly enhances toughness when aligned in an ordered arrangement [13,14,15]. He et al. prepared montmorillonite (MMT)-reinforced PLA/Polybutylene adipate terephthalate (PBAT) composites and systematically investigated their structure–property relationships [15]. The results indicated that MMT tends to distribute at the PLA/PBAT interface, and organically modified MMT can undergo esterification with PLA/PBAT, leading to good interfacial compatibility and dispersion. Mechanical tests further confirmed that the incorporation of MMT improved both the tensile elongation at break and impact toughness of the composites. Furthermore, the composite’s performance is closely linked with the dispersion and alignment of fillers [16]. Therefore, adopting appropriate alignment control techniques to precisely orient the arrangement of two-dimensional fillers is crucial for fully exploiting the performance potential of composites.

Currently, the application of external fields, such as electric fields [17,18], magnetic fields [19,20], or mechanical force fields [21,22,23], can induce the directional alignment of two-dimensional fillers in polymer melts and promote the formation of macroscopically continuous structures. After cooling and solidification, composites with well-ordered orientation can be obtained. Compared with electric or magnetic field induction, mechanical force field-based technology offers advantages such as simple principles, convenient operation, and a wide range of field strength adjustability [24,25]. It is particularly suitable for the continuous production of profiles, such as films, rods, and microtubes, and is characterized by high production efficiency and well-established process maturity, making it an ideal approach for preparing two-dimensional filler-reinforced composites [26,27]. At present, most polymer microtubes are manufactured via melt extrusion processes. In conventional extrusion, the combined actions of screw conveying, die shear, and haul-off stretching create a unidirectional axial mechanical force field, which promotes the preferential alignment of two-dimensional fillers along the tube axis. However, two-dimensional fillers possess two rotational degrees of freedom. Under a single axial force field, due to the lack of constraints in other directions, the fillers undergo free rotation along the axial direction [28,29,30]. This results in preferential orientation in the longitudinal cross-section along the axis, while remaining randomly distributed in the radial cross-section. This orientation inhomogeneity leads to lower hoop performance compared to axial performance in PLA microtubes, severely limiting their application in multi-directional loading scenarios. Therefore, constructing a multi-dimensional force field to effectively regulate and enhance hoop strength has become a key strategy to overcoming the performance limitations of PLA microtubes and expanding their practical applications.

In response to the limitations of orientation control in conventional extrusion, this study introduces a rotational extrusion device that superimposes a rotational force field on the traditional axial force field. By adjusting the rotational speed to regulate hoop stress during processing, the alignment of two-dimensional sheet-like fillers along the hoop direction is induced, and the synergistic effects on the axial and hoop mechanical properties of composite microtubes are systematically investigated. Graphene oxide (GO) was selected as the reinforcement, due to its favorable dispersion stemming from active surface groups, while a biodegradable PLA/PBAT blend served as the polymer matrix. Specifically, 1 wt% GO nanosheets were incorporated as the two-dimensional reinforcement phase, along with 2 wt% ADR 4468 epoxy chain extender to improve interfacial compatibility, yielding GO-reinforced PLA/PBAT nanocomposite microtubes. Through precise control of the rotational speed, a concentric, interlocked alignment network of fillers along the circumferential direction was successfully constructed, while the axial orientation of GO remained preserved. This dual-scale structural control ensures the retention of axial mechanical strength, together with a marked enhancement in hoop mechanical performance, resulting in nanocomposite microtubes with superior and balanced directional properties. This work, thus, provides a viable and efficient strategy for the industrial manufacturing of high-performance composite microtubes.

## 2. Experimental Section

### 2.1. Materials

Poly(lactic acid) (PLA), grade 4032D, was provided by Nature Works (Plymouth, MN, USA). It has a density of 1.24 g/cm^3^ and a melt flow index of 7 g/10 min (210 °C, 2.16 kg). Polybutylene adipate terephthalate (PBAT) with grade TH801T, produced by Xinjiang Blue Ridge Tunhe Technology Co., Ltd. (China), has a density of 1.21 g/cm^3^ and a melt flow rate of 3.6 g/10 min (190 °C, 2.16 kg). Graphene Oxide (GO), model SE2430, has a tap density of 0.7–1.2 g/cm^3^ and a particle size of 15–30 μm, produced by China Changzhou Sixth Element Materials Technology Co., Ltd. (China). It possesses a two-dimensional geometry and is functionalized with surface oxygen-containing groups, primarily hydroxyl (-OH) and carboxyl (-COOH) groups. The epoxy-based chain extender Joncryl^®^ ADR 4468S, produced by BASF (Germany), has a molecular weight of 6800 g/mol and an epoxy equivalent weight of 285 g/mol. It has epoxy functional groups, which can react with polymer chain ends, providing the in situ compatibilization necessary for stabilizing the blend morphology during the complex flow.

### 2.2. Sample Preparation

**Figure 1:**
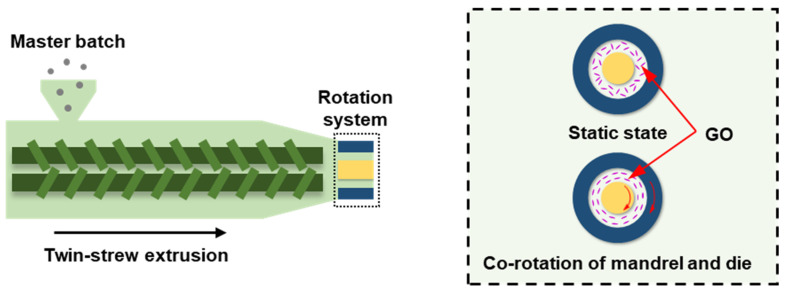
Schematics and mechanisms of the preparation of composite microtubes via rotating extrusion.

All raw materials were uniformly blended in a HAPRO rheometer (RM-200A, China Harbin HAPO Electric Technology Co., Ltd.) to prepare the master batch. For master batch, the PLA/PBAT mass ratio was maintained at 8:2, with the addition of 1 wt% GO and 2 wt% ADR (relative to PLA/PBAT blend). The raw materials were first dried in a vacuum oven at 60 °C for 12 h. The mixing process was conducted at a screw speed of 35 rpm and a processing temperature of 180 °C for a duration of 5 min to ensure homogeneous dispersion. The obtained master batch was extruded into the composite tube via self-designed rotation extrusion equipment with rotational mandrel and die, and this special equipment has been used in our previous work [31]. In terms of temperature, the extrusion system zones were maintained at 120 °C, 165 °C, 175 °C, and 175 °C, with the rotational extrusion die head set at 170 °C and 165 °C. To further clarify the experimental system, Figure 1 illustrates the sample preparation process and the internal structural evolution. In this case, the polymer melts were extruded axially, with a screw speed of 15 rpm and axial pulling speed of 20 cm/min. The rotational speeds of the mandrel and die were synchronized and set at 0, 10, 20, and 30 rpm to study the effect of rotational speed on the performance of composite tube. For example, the rotation of the mandrel and die at 30 rpm induced a circumferential flow of the polymer melt in the gap, resulting in a circumferential stress rate exceeding 10 s^−1^. This circumferential stress, coupled with the axial stress, led to a biaxial stress state. The prepared microtube had an outer diameter of 4 mm, with a wall thickness of 0.6 mm. The samples were labeled as Rx, where x corresponded to their rotational speeds.

### 2.3. Characterization and Measurement

The scanning electron microscope (FEI Inspect F-SEM, USA) was employed to examine the fracture surfaces, etched morphologies, and cryo-fractured cross-sections of the composite microtubes. Prior to SEM observation, all samples were sputter-coated with gold to enhance conductivity, and the microscope was operated at an acceleration voltage of 5 kV. In the selective etching process used to reveal the GO network, the fractured samples were immersed in a pre-prepared etching solution (consisting of deionized water and methanol in a 1:1 volume ratio, with 0.05 mol/L NaOH added) and allowed to etch statically at 25 °C for 12 h. After etching, the samples were ultrasonically cleaned in deionized water for 15 min to remove residue.

The two-dimensional small-angle X-ray scattering (2D-SAXS) patterns of the samples were measured using a Xeucs 2.0 system (GeniX3D Cu ULD, Xenocs SA, France) to characterize the orientation variations along the circumferential direction of the composite microtubes. The experiments were performed with scatter-free collimating slits using a multilayer-confocal Cu Kα X-ray source (wavelength: 0.154 nm), with a sample-to-detector distance of 2474 mm. The sheet specimens were sectioned along the circumferential direction of the microtubes, carefully polished with sandpaper, and the cross section was exposed orthogonally to X-ray beam. When the platelet-shaped fillers were randomly distributed, an isotropic diffuse scattering pattern was observed. In contrast, when the fillers exhibited preferential alignment along the hoop direction, with their planar surface perpendicular to the radial direction of the tube, an anisotropic scattering pattern was generated. A schematic illustrating the alignment pattern from the viewing direction, based on SAXS images obtained from the cross sections, is shown in Figure S2. The orientation degree (f) was calculated from the integrated one-dimensional azimuthal curves of the 2D-SAXS patterns using Herman’s orientation parameter, with the f-value calculated according to the following equation [32]:

(1) $$f=\frac{3\left\langle \cos^{2}\phi \right\rangle -1}{2}$$

Therein, $\left\langle \cos^{2}\phi \right\rangle \;$ was obtained from the azimuthal intensity profile, as follows:

(2) $$\left\langle \cos^{2}\phi \right\rangle =\frac{\int_{0}^{\pi ∕2}I\left(\phi \right)\sin\phi \left\langle \cos^{2}\phi \right\rangle d\phi }{\int_{0}^{\pi ∕2}I\left(\phi \right)\sin\phi d\phi }$$

where $I\left(\phi \right)$ represents the scattering intensity at azimuthal angle ϕ. When the fillers are isotropically aligned, f=0; when the fillers are perfectly aligned parallel to the direction of the hoop stress, f=1.

The crystallization behavior of the composite microtubes was characterized using a Differential Scanning Calorimeter (DSCQ20, TA Instruments, New Castle, DE, USA). A sample weighing 3–5 mg was cut from the microtube and placed in a crucible. Under a nitrogen atmosphere, the sample was heated from 30 °C to 200 °C at a rate of 10 °C/min, and the first heating curve was recorded. The crystallinity of the sample can be calculated using the following equation [33,34]:

(3) $$X_{C}=\frac{\Delta H_{m}-{\Delta H}_{cc}}{\Delta H_{0}\times wf}\times 100\%\;$$

where $\Delta H_{m}$ is the melting enthalpy of the sample, ${\Delta H}_{cc}$ is the cold crystallization enthalpy of the sample, $\Delta H_{0}$ is the melting enthalpy of a fully crystalline sample, and wf represents mass content of the PLA component in the mixture. For PLA, $\Delta H_{0}$ = 93.7 J/g [34].

The radial cyclic compression properties of composite microtubes were investigated using an electronic universal testing machine (BOSS 3220 SERIES II, USA). Samples with uniform dimensions (4 mm in length) were cut from the microtubes. Each specimen was subjected to 100 cycles of compression at a fixed strain of 5% and a frequency of 1 Hz, using a sinusoidal waveform. The microtubes were placed parallel and centered on the compression platform. The equation for calculating compressive stress is as follows [35]:

(4) $$K=\frac{F\left(D-d\right)}{LD^{2}}$$

where L is the sample length, F is the compressive load, and D and d are the outer and inner diameters of the tube, respectively. Each sample was tested 10 times to minimize errors.

The bending properties of composite microtubes were investigated using a universal testing machine (DWD-10 KN, Sichuan Dexiang Technology Innovation Instrument Co., Ltd., China). Microtubes were cut into uniform samples measuring 90 mm in length. The measurements were performed in three-point bending mode with a span of 64 mm and a bending indenter speed of 5 mm/min. Ten measurements were conducted for each sample to minimize experimental error.

The tensile properties of composite microtubes were investigated using a universal testing machine (Reger RGL-10, Shenzhen Reger Company, China). The microtubes were cut into uniform specimens with a length of 40 mm. Prior to testing, matching cylindrical wooden sticks were inserted into both ends of each tube to prevent failure at the clamping points. A 1 kN load cell was used, with a tensile rate of 2 mm/min and a gauge length of 20 mm. Ten measurements were performed for each sample to minimize experimental error.

The impact performance of composite microtubes at low temperature was investigated using a pendulum impact testing machine (ZBC-4B, Shenzhen Xinsansi Metrology Technology Co., Ltd., China). The microtubes were cut into uniform specimens with a length of 110 mm. The samples were immersed in liquid nitrogen until their core temperature reached −10 °C. They were then rapidly removed and immediately impacted by the pendulum hammer within a very short timeframe to complete the fracture test before significant temperature change could occur. A pendulum mass of 1 kg was used. Ten measurements were performed for each sample to minimize experimental error.

## 3. Results and Discussion

### 3.1. GO Alignment in Composite Microtubes

The physical and chemical properties of graphene oxide (GO), such as particle size, layer thickness, oxygen content, and reactivity, play crucial roles in determining the final performance of the nanocomposite microtubes. Therefore, the fundamental characteristics of GO were first characterized. Figure S1 displays the SEM morphology of GO, the corresponding elemental distribution map of oxygen, the XPS O 1s spectrum, and the particle size distribution. As shown in Figure S1a, the GO nanosheets exhibit a typical multi-layered flake structure, with an in-plane width ranging from approximately 10 to 20 μm. The corresponding EDS image (Figure S1b) illustrates the distribution of oxygen, where it is clearly observed that the yellow signal points representing oxygen are predominantly concentrated in the areas corresponding to the GO nanosheets, indicating the presence of abundant oxygen-containing functional groups. The XPS spectrum (Figure S1c) shows a distinct O 1s peak, further confirming the high oxygen content in GO. Particle size analysis (Figure S1d) reveals that the average particle size of GO is approximately 22.98 μm, with the particle size distribution concentrated within the 10–100 μm range, demonstrating that the GO nanosheets have uniform dimensions, which facilitates their effective dispersion and alignment within the polymer matrix.

**Figure 2:**
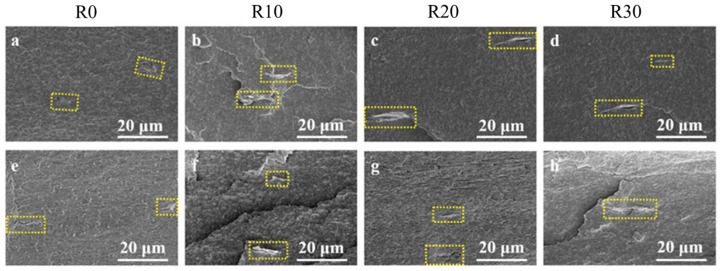
Radial direction (**a**–**d**) and axial direction (**e**–**h**) SEM images of the GO-filled composite microtubes with different rotational speeds.

The alignment evolution of GO in composite microtubes with increasing rotational speed was investigated through SEM of both radial (Figure 2a–d) and axial cross-sections (Figure 2e–h). In the radial cross-sections, the GO orientation progressively aligns with increasing rotational speed, as highlighted by the yellow markers. This demonstrates that the enhanced hoop stress, resulting from the rotation, effectively promotes the formation of unidirectional GO alignment along the circumferential direction. In contrast, the axial cross-sections reveal consistently well-aligned GO structures regardless of rotational speed, indicating that the intensified rotational force field did not significantly disturb the axial stress field, thereby maintaining excellent GO alignment along the axial direction. These results collectively demonstrate that the synergy between the axial stress field and the rotation-induced hoop stress field is key to inducing well-defined GO orientation in both directions, thereby enhancing overall orientation regularity.

**Figure 3:**
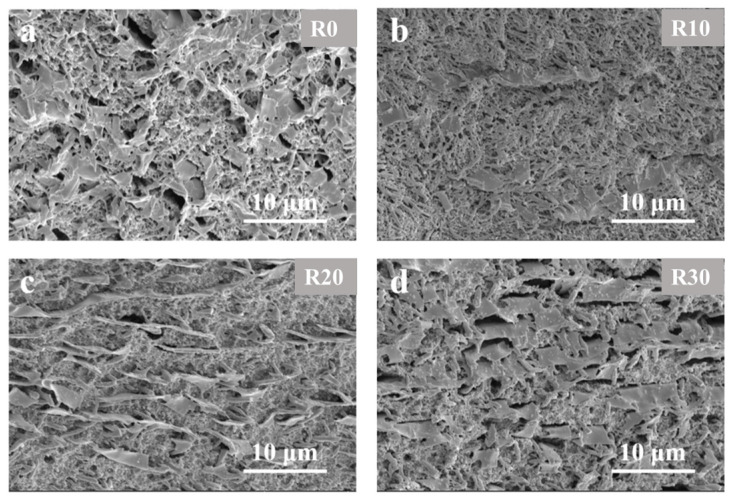
Radial direction SEM images of selectively etched morphology of GO-filled composite microtubes with different rotational speeds. (**a**) R0; (**b**) R10; (**c**) R20; (**d**) R30.

To eliminate the interference from the polymer matrix and better observe the alignment of GO under varying rotational stresses, selective etching was performed on the radial cross-sections of the composite microtubes. Figure 3a–d show the distribution and orientation of GO in the radial cross-sections of the microtubes at rotational speeds of 0 rpm, 10 rpm, 20 rpm, and 30 rpm, respectively. It can be seen that, under conventional extrusion conditions, the uniaxial stress alone cannot induce an ordered circumferential orientation of GO, and no continuous alignment of GO along the circumferential direction is observed. After introducing a rotational speed of 10 rpm, the GO alignment begins to exhibit a more regular pattern. As shown in Figure 3b, some GO nanosheets are oriented along the circumferential direction, although a continuous linear alignment has not yet formed. When the rotational speed increases to 20 rpm (Figure 3c) or 30 rpm (Figure 3d), a “tip-to-tip” connection morphology of GO nanosheets becomes visible, with a well-developed continuous alignment. This phenomenon indicates that, under high rotation-induced hoop stress, GO nanosheets gradually form a concentric, interconnected alignment network within the microtube. This highly interconnected nanofiller network is key to improving the mechanical, conductive, and barrier properties of the composite microtubes, serving as a fundamental basis for their high-performance optimization [36,37,38].

**Figure 4:**
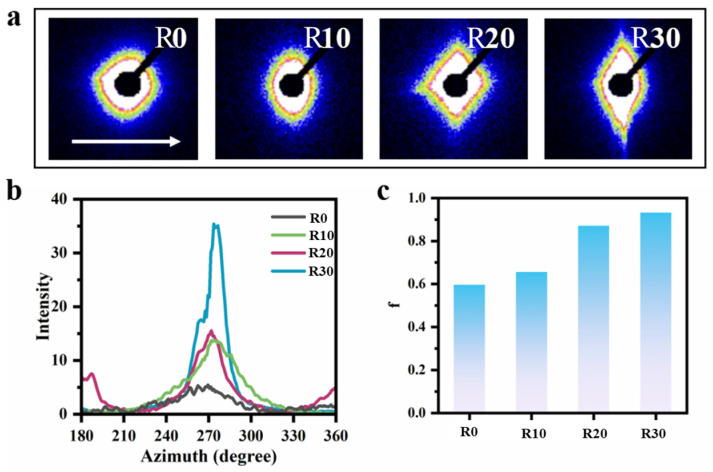
2D-SAXS spectra of the GO-filled composite microtubes with different rotating speeds. The arrow indicates the hoop direction (**a**); the corresponding azimuth angle curves (**b**) and the orientational degree (f), as estimated by the Herman’s orientational parameter (**c**).

Furthermore, the circumferential orientation of GO within the composite microtubes was characterized using 2D small-angle X-ray scattering (SAXS) patterns, with the corresponding one-dimensional azimuthal intensity profiles and orientation degree (f) values statistically analyzed, as shown in Figure 4. It is clearly observed in Figure 4a that, as the rotation speed increases, the scattering pattern of the composite microtubes gradually transitions from a circular to an elliptical, and eventually to a rhombus, shape. The orientation direction aligns with hoop stress, indicating that the rotation-induced hoop force successfully induces the ordered alignment of GO along the circumferential direction. As shown in Figure 4b,c, the f values of the azimuthal profiles increase progressively with rising rotation speed, demonstrating that the degree of GO orientation along the circumferential direction improves accordingly, reaching its maximum at 30 rpm.

### 3.2. Crystal Structure of Composite Microtubes

**Figure 5:**
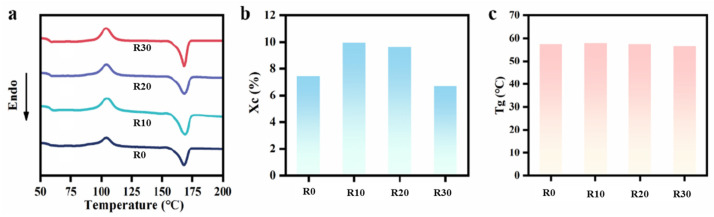
DSC heating curves of the GO-filled composite microtubes with different rotating speeds (**a**) and the corresponding values of Xc (**b**) and Tg (**c**).

The crystal structure of the composite microtubes under different rotational speeds was characterized using DSC, as shown in Figure 5. The results indicate that the rotation-induced hoop stress field slightly influences the crystallinity of the nanocomposite microtubes (Figure 5b), while it does not alter their glass transition temperature (Figure 5c). The crystallinity of the four composite microtubes fell within the range of 8% to 10%, suggesting that rotation extrusion did not significantly affect the crystal structure.

### 3.3. Mechanical Properties of Composite Microtubes

#### 3.3.1. Hoop Mechanical Properties

The structural characterization results confirm that the biaxial stress field generated by the rotational extrusion equipment successfully induces continuously aligned structures of GO nanosheets within the composite microtubes. Compared to conventional extrusion, this strategy results in significantly improved regularity and continuity of filler alignment along the circumferential direction. This optimized microstructure allows for the full exploitation of the mechanical advantages of two-dimensional nanosheets, enhancing the hoop stress-bearing capacity of the composite microtubes and leading to superior overall mechanical performance. Building upon this foundation, the hoop mechanical properties of the composite microtubes with GO were systematically characterized.

**Figure 6:**
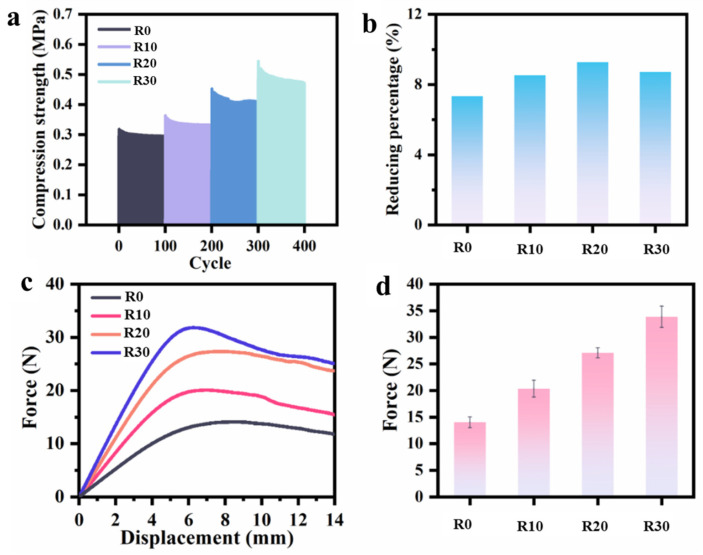
Radial compressing properties of the GO-filled composite microtubes with different rotating speeds (**a**) and the corresponding reducing percentage of 100th cycle compared with 1st cycle (**b**); bending properties of the GO-filled composite microtubes with different rotating speeds (**c**) and the corresponding maximum force values during bending tests (**d**).

Radial cyclic compression tests were performed on the composite microtubes at different rotational speeds under a frequency of 1 Hz for 100 cycles, with the compression displacement fixed at 5% of the tube diameter. As observed in Figure 6a, the compressive strength of the composite microtubes shows a significant enhancement with increasing rotational speed. When the rotational speed reaches 30 rpm, the maximum compressive strength reaches 0.54 MPa, which represents an approximately 69% increase compared to microtubes prepared by conventional extrusion (0.32 MPa). This result verifies the initial hypothesis that the rotational extrusion process enhances hoop mechanical properties by regulating filler orientation. Due to the unique anisotropic structure of two-dimensional fillers, whose in-plane mechanical properties far exceed their out-of-plane properties [39,40], their alignment architecture has a profound impact on the composite’s performance. When the nanosheets are aligned perpendicular to the compression direction, the filler–matrix interface can efficiently transfer stress along the filler’s in-plane direction. Furthermore, the GO nanosheets, aligned circumferentially (i.e., perpendicular to the radial direction), can effectively suppress the propagation of local cracks. This structural design achieves the goal of performance optimization. Figure 6b quantifies the reduction percentage of the compressive strength at the 100th cycle compared to the 1st cycle. The results show that the reduction rates for all four samples fall within the range of 7–9%, indicating that the GO-reinforced PLA/PBAT composite microtubes possess excellent resistance to radial compressive deformation and can maintain good mechanical performance and dimensional stability in practical applications. Figure 6c,d present the bending properties of the composite microtubes. It can be observed that the maximum force under bending load gradually increases with higher rotational speeds. Compared to microtubes produced by conventional extrusion, the maximum bending force of the rotationally extruded composite microtubes at 30 rpm increases by approximately 133%, following a trend similar to the cyclic compressive strength. This enhancement is attributed to the synergistic effect of the axial stress and the rotation-induced hoop stress at elevated rotational speeds, which promotes the formation of a layered GO structure aligned perpendicular to the radial direction. This specific architecture enables the microtubes to withstand greater bending loads.

#### 3.3.2. Axial Mechanical Properties

**Figure 7:**
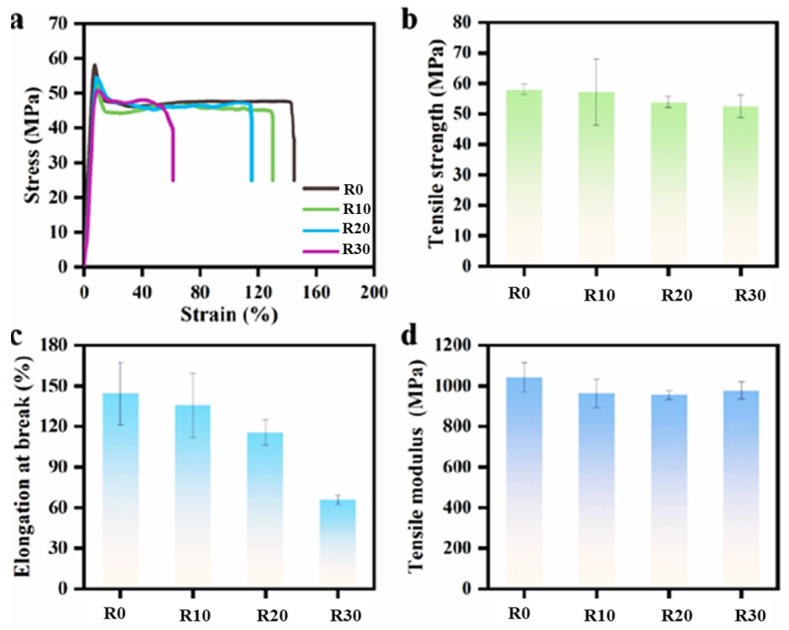
The stress–strain curves of GO-filled composite microtubes with different rotating speeds (**a**) and the corresponding tensile strength (**b**), elongation at break (**c**), and tensile modulus (**d**).

The axial mechanical properties of the nanocomposite microtubes were tested. As shown in Figure 7a, the tensile stress–strain curves of all composite microtube samples exhibit characteristics of ductile fracture. The elongation at break initially decreases slowly, but then drops significantly, to approximately 67% at 30 rpm (Figure 7c). This phenomenon can be explained by the fracture mechanism under axial tensile loading. During testing, the relatively weak interfacial adhesion between the GO and the polymer matrix leads to initial debonding and hole formation at the interfaces. As the tensile displacement increases, these micro-defects propagate along the circumferential direction of the tube, eventually coalescing into macroscopic cracks that cause material failure. For microtubes produced by conventional extrusion, the GO nanosheets exhibit a disordered arrangement within the circumferential plane. Some GO nanosheets, oriented at angles to or perpendicular to the circumference, can obstruct the linear propagation of micro-cracks along the circumferential direction. This obstruction promotes energy dissipation mechanisms, such as molecular chain slippage and shear band formation. In contrast, for rotationally extruded microtubes, especially those subjected to higher rotational stress, the majority of GO nanosheets form a highly aligned, uniform circular structure along the circumferential direction. This uniform alignment creates a continuous and unobstructed pathway at the interfaces, allowing the debonding defects and micro-cracks to propagate rapidly along this “preferential path” in the circumferential direction. However, with increasing rotational speed, both the tensile strength and Young’s modulus of the microtubes show slight decreases, but are still maintained at high values (Figure 7b,d), as the axial filler orientation is not compromised by the applied rotational speed. This finding provides critical insight for achieving a balanced optimization between the axial and hoop mechanical properties in composite microtubes.

#### 3.3.3. Low-Temperature Impact Performance

**Figure 8:**
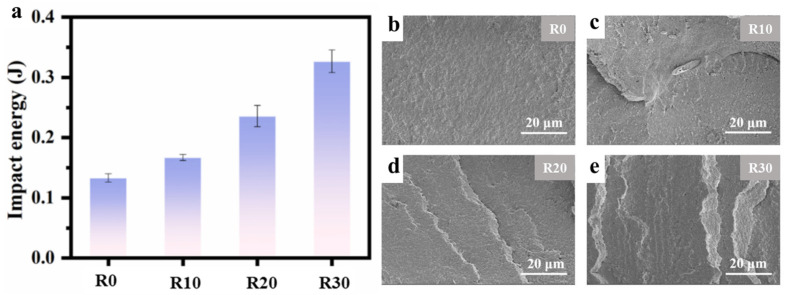
Impact energy of the GO-filled composite microtubes with different rotating speeds (**a**); SEM photographs of the impact-fractured surfaces of the GO-filled composite microtubes with different rotating speeds (**b**–**e**).

To investigate the contribution of GO nanosheet alignment evolution to impact performance, the low-temperature impact properties of the composite microtubes were evaluated. As shown in Figure 8a, the sample prepared at a rotational speed of 30 rpm achieves an impact energy as high as 0.33 J, representing an approximately 146% increase compared to the sample produced by conventional extrusion. This improvement is attributed to the formation of a macroscopic and continuous “barrier” by the GO nanosheets aligned perpendicular to the radial direction. The continuous, circumferentially aligned structure of GO nanosheets effectively inhibits the propagation of local cracks and dissipates more instantaneous impact energy during the impact process, thereby enhancing the impact toughness of the sample. The fracture morphology of the low-temperature impact surfaces observed via SEM provides direct evidence of how filler orientation influences the fracture mechanism. As shown in Figure 8b, the impact fracture path in the conventionally extruded sample is smooth and flat, aligning closely with the loading direction. This is due to the disordered distribution of GO nanosheets along the hoop direction, which offers little resistance to crack propagation. In contrast, as the rotational speed increases, the impact fracture surfaces become progressively rougher and more rugged, with an increase in step-like features (Figure 8c–e). The elevated hoop stress at higher rotational speeds drives the GO nanosheets to form an oriented structure perpendicular to the radial direction. This creates a robust “brick-and-mortar”-like barrier, with the layered GO nanosheets embedded in the polymer matrix. According to the crack propagation mechanism, local cracks must repeatedly traverse these vertically aligned nanosheets to cause fracture, resulting in the observed stepped fracture morphology.

## 4. Conclusions

In this study, a rotational extrusion strategy is successfully developed to construct a biaxial stress field, which enables the precise tailoring of GO orientation in composite microtubes. By superimposing a rotational force field onto the conventional axial flow, a concentric and interconnected GO alignment network was induced along the circumferential direction without compromising the pre-existing axial filler orientation. This architecturally controlled structure significantly enhances the hoop mechanical properties, including compressive strength, bending properties, and low-temperature impact properties, owing to the formation of a continuous “brick-and-mortar”-like barrier that effectively transfers and distributes stress. Importantly, the axial tensile strength and modulus are well maintained due to the preserved axial alignment. This work, thus, provides a simple, efficient, and scalable processing route to fabricate high-performance composite microtubes with tunable and balanced directional properties, holding promising potential for industrial applications, such as medical tubing and lightweight structural components.

## Data Availability

The original contributions presented in this study are included in the article/Supplementary Materials. Further inquiries can be directed to the corresponding author.

## Supplementary Materials

The following supporting information can be downloaded at: https://www.mdpi.com/article/10.3390/polym18020273/s1.
