# Supplementary material for: Constructing a Concentric GO Network via Rotational Extrusion for Synergistic Axial–Hoop Mechanics in Polymer Microtubes
Source: Polymers (Basel). 2026 Jan 20;18(2):273. doi: 10.3390/polym18020273 (PMC12845656; doi:10.3390/polym18020273)
Supplement: Supplementary file 1 [file polymers-18-00273-s001.zip › polymers-4081523-supplementary.pdf]

Supporting Information

Constructing a Concentric GO Network via Rotational Extrusion for Synergistic Axial–Hoop Mechanics in Polymer Microtubes

Wenyan Wang [1,2,3], Wen Liang [1], Guanxi Zhao [3], Rui Han [2] and Min Nie [1,*]

[1] *State Key Laboratory of Advanced Polymer Materials, Polymer Research Institute of Sichuan University, Chengdu 610065, China; wwyandmmy@163.com (W.W.)*

[2] *School of Materials Science and Engineering, Key Laboratory of Materials and Surface Technology (Ministry of Education), Engineering Research Center of Intelligent Air-Ground Integration Vehicle and Control, Xihua University, Chengdu 610039, China*

[3] *Sichuan Special Equipment Inspection Institute, Technology Innovation Center of Hydrogen Storage-Transportation and Fueling Equipments, State Administration for Market Regulation, Chengdu 610000, China*

* *Correspondence: poly.nie@gmail.com*

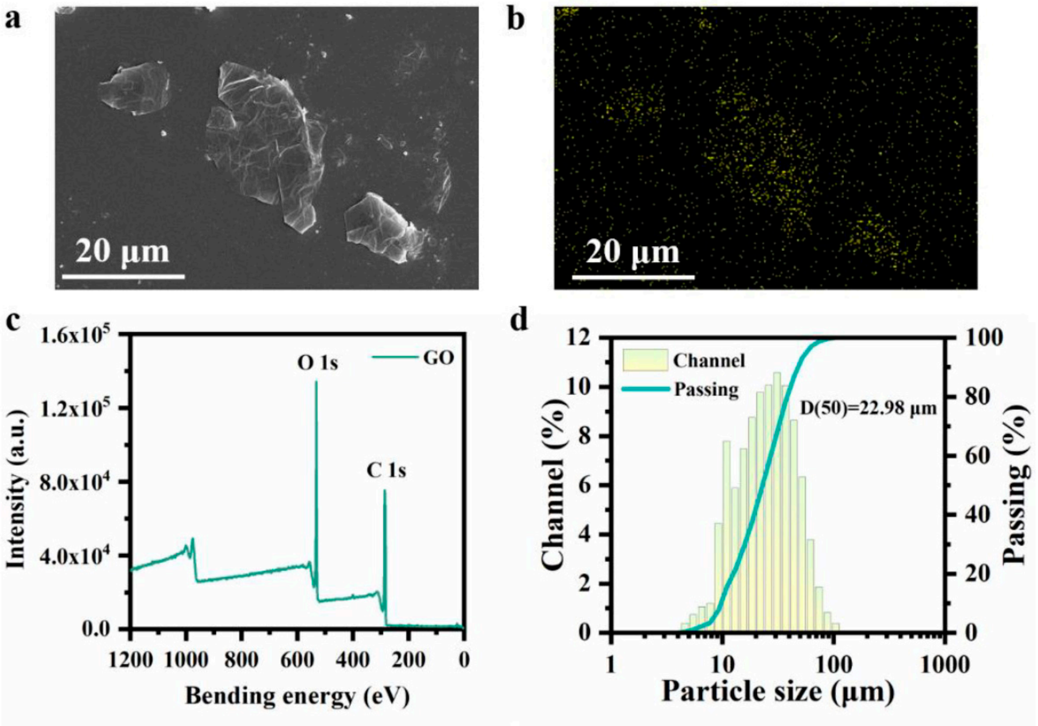

Figure S1. SEM image of the GO nanosheet (a) and corresponding distribution of oxygen from EDS spectra of the GO nanosheet (b); XPS image of GO nanosheets (c); Size distribution of GO nanosheets.

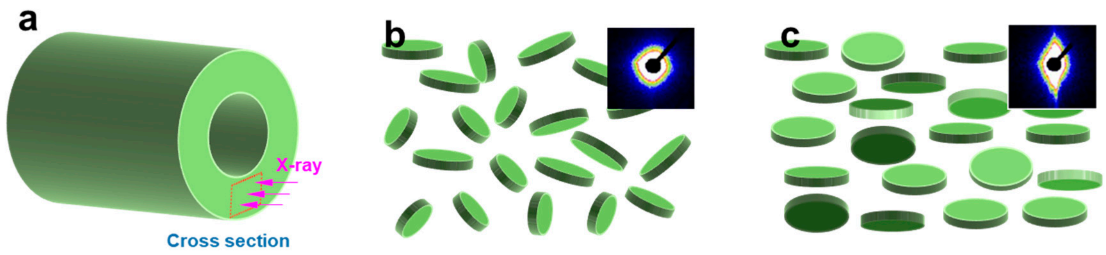

Figure S2 Schematic illustration of SAXS investigation in the cross sections (a); The alignment pattern at the view direction based on the SAXS images with the insets showing SAXS images of isotropic alignment (b) and preferential alignment (c).
